# Effects of a ZnCuO-Nanocoated Ti-6Al-4V Surface on Bacterial and Host Cells

**DOI:** 10.3390/ma15072514

**Published:** 2022-03-29

**Authors:** Kamal Dabbah, Ilana Perelshtein, Aharon Gedanken, Yael Houri-Haddad, Osnat Feuerstein

**Affiliations:** 1 Department of Prosthodontics, Hadassah Medical Center, Faculty of Dental Medicine, The Hebrew University of Jerusalem, Jerusalem 9112001, Israel; kamal.dabbah@gmail.com (K.D.); yaelho@ekmd.huji.ac.il (Y.H.-H.); 2 Department of Chemistry, Bar-Ilan Institute of Nanotechnology and Advanced Materials, Bar-Ilan University, Ramat-Gan 5290002, Israel; ilana.perelshtein@biu.ac.il (I.P.); aharon.gedanken@biu.ac.il (A.G.)

**Keywords:** antimicrobials/antimicrobial resistance, biofilm(s), dental implant(s), immunity, osteoblast(s), macrophage(s), peri-implant infection(s), zinc oxide, biofilm model

## Abstract

This study aims to investigate the effects of a novel ZnCuO nanoparticle coating for dental implants—versus those of conventional titanium surfaces—on bacteria and host cells. A multispecies biofilm composed of *Streptococcus sanguinis*, *Actinomyces naeslundii*, *Porphyromonas gingivalis*, and *Fusobacterium nucleatum* was grown for 14 days on various titanium discs: machined, sandblasted, sandblasted and acid-etched (SLA), ZnCuO-coated, and hydroxyapatite discs. Bacterial species were quantified with qPCR, and their viability was examined via confocal microscopy. Osteoblast-like and macrophage-like cells grown on the various discs for 48 h were examined for proliferation using an XTT assay, and for activity using ALP and TNF-α assays. The CSLM revealed more dead bacteria in biofilms grown on titanium than on hydroxyapatite, and less on sandblasted than on machined and ZnCuO-coated surfaces, with the latter showing a significant decrease in all four biofilm species. The osteoblast-like cells showed increased proliferation on all of the titanium surfaces, with higher activity on the ZnCuO-coated and sandblasted discs. The macrophage-like cells showed higher proliferation on the hydroxyapatite and sandblasted discs, and lower activity on the SLA and ZnCuO-coated discs. The ZnCuO-coated titanium has anti-biofilm characteristics with desired effects on host cells, thus representing a promising candidate in the complex battle against peri-implantitis.

## 1. Introduction

The success of dental implants depends on the integration of the implant with the bone and mucosal connective tissue, as well as the absence of inflammation and infection in the surrounding tissues [1]. Although many studies have been carried out to improve the surface modification of titanium implants [2,3], bacterial accumulation as biofilms on dental implant surfaces induces inflammatory changes in the tissues surrounding the implant. Biofilm composition and virulence, in conjunction with the immune–inflammatory response, can cause peri-implantitis and peri-implant mucositis [4]. As some 10% of dental implant late failure is attributable to peri-implantitis and chronic inflammation in the supporting tissues [5,6], it is important to evaluate the effects of surface modification of implants on biofilm characteristics [7], as well as on the surrounding tissues.

Biofilm formation on different substrata in the oral cavity begins with the adhesion of primary colonizers such as *Streptococcus* spp. and *Actinomyces* spp. via interaction of the bacterial wall with the salivary film formed on the substrate [8,9]. The early colonizers provide the link for the establishment of secondary colonizers by co-adhesion and co-aggregation of the microorganisms, promoting an increase in cell number and biofilm tridimensional organization [10]. The composition of the pathogenic biofilm present in peri-implantitis is similar to that in periodontitis [11]. Although the oral cavity provides a similar ecological environment for the composed biofilm on the surfaces of titanium implants and natural teeth [12], surface characteristics—such as material composition and roughness—may affect the established biofilms [13].

The development of implant surfaces that further osseointegration in addition to bacterial inhibition is important to the clinical success of implants [14]. Surface changes in nanotopography exert their effects at the physical, chemical, and biological levels [15], resulting in increased adhesion of osteogenic cells [16], and thereby potentially promoting osseointegration. Metal nanoparticles (NPs), metals, and their oxides have been shown to have strong antimicrobial activity, with no drug resistance known so far [17]; thus, they are suggested for dental applications, such as restorative materials, acrylic denture base resins, orthodontic appliances, oral hygiene aids, and dental implants [18]. The antibacterial properties of coated Ti-6Al-4V alloy with Cu and Zn metals or oxides were obtained by different methods, such as electrohydrodynamic spraying [19], a plasma-immersion ion implantation system (PIII) [20], and one-step micro-arc oxidation (MAO) [21].

The sonochemical coating technique was proven to be a highly effective technique due to the fact that the coated particles are strongly adhered to the surface. Textiles that were coated via the sonochemical technique with antibacterial nanoparticles were washed for 65 washing cycles, after which the particles were found on the surface and the antibacterial properties were retained [22]. Recently, a new zinc-doped CuO nanocomposite with enhanced anti-biocidal properties was sonochemically synthesized, and its antibacterial activity was proven to be 10,000–100,000x more effective than ZnO or CuO alone [23]. Moreover, the coating and synthesis takes place via a one-step process, where the ZnCuO is formed and subsequently deposited on the surface. The anti-biofilm activity of these ZnCuO-coated surfaces on teeth and artificial teeth was demonstrated against *Streptococcus mutans* [24,25].

Therefore, we hypothesized that the topography and chemical modification of titanium surfaces by sonochemical coating of ZnCuO nanoparticles may have anti-biofilm and antibacterial effects on oral bacteria in biofilms, and could affect the viability and activity of the host cells. Hopefully, this would influence the inflammatory response and the osseointegration of ZnCuO-coated dental implants. The aim of this study was to investigate the effects of new ZnCuO-coated nanoparticle titanium surfaces versus conventional ones on bacterial viability and composition in a multispecies oral biofilm model, and on the viability and metabolic activity of the host osteoblast-like and macrophage-like cells, when cultured on the various hydroxyapatite surfaces.

## 2. Materials and Methods

### 2.1. Characterization of the ZnCuO Coating

The content of copper and zinc on the coated surface was evaluated by inductively coupled plasma (ICP), using a Spectro ICO-Blue Tl optical emission spectrometer (GmbH, Kleve, Germany). The preparation for the ICP measurements was as follows: a weighed piece of disc was inserted into 10 mL of 0.5 M nitric acid for the dissolution of the zinc–copper oxide particles from the surface. The solution was boiled for 15 min, and then 15 mL of double-distilled water was added, and the solution was boiled for another 15 min. At the end, the volume of the solution was adjusted to 50 mL. The crystalline structure of the Zn–CuO particles was studied by X-ray diffraction (XRD) using a Bruker D8 Advance X-107 X-ray diffractometer (Bruker, Billerica, MA, USA), with Cu Kα (λ = 1.5418 Å) as a source. The morphology and size of the particles on the disc surface were studied using high-resolution scanning electron microscopy (HRSEM) (FEI, Lausanne, Switzerland) with a Magellan FEI microscope, at an accelerating voltage over the range of 5–15 kV. To improve the quality of the images, the samples were coated with a carbon layer by sputtering in a rarefied atmosphere of argon at 0.1–0.2 mbar, by means of an Emitech K550 Sputter Coater.

### 2.2. Sample Preparation

Titanium alloy (Ti 6AI-4V) discs, 5 mm in diameter x 1 mm thickness, were prepared with machined, sandblasted, and sandblasted + acid-etched (SLA) (Alpha Bio, Petah Tikva, Israel) surfaces. Some of the machined surface discs were coated with ZnCuO nanoparticles using a sonochemical method described in detail by Malka et al. (2013) [23]. Briefly, zinc and copper acetates served as precursors. The titanium discs were placed in a solution containing zinc and copper at a 1:3 molar ratio, and sonochemical irradiation was initiated with a high-intensity ultrasonic Ti horn (Sonics & Materials, Newtown, CT, USA). After a temperature of 60 °C was reached, an aqueous solution of ammonium hydroxide (28%) was injected into the reaction cell to adjust the pH to ~8, and the sonochemical deposition was allowed to proceed for 1 h. Hydroxyapatite discs 5 mm in diameter × 2 mm thickness (Clarkson Chromatography Product Inc., Williamsport, PA, USA), along with machined titanium discs, served as controls.

### 2.3. Multispecies Biofilm Model

A four-species biofilm—consisting of the oral bacteria *S. sanguis NC02863*, *A. naeslundii 17233*, *P. gingivalis TACC33277*, and *F. nucleatum PK1594*—was grown in 96-well culture plates on disc samples in gingival crevicular fluid (GCF)-simulating medium [26]. Human saliva (Helsinki board approval HMO052511) diluted 1:4 in double-distilled water (DDW) [27] was inoculated on disc samples, which were incubated for 30 min at 37 °C to simulate the acquired pellicle. The discs were washed with phosphate-buffered saline (PBS), a suspension of *S. sanguinis* and *A. naeslundii* (1:1 ratio in a total volume of 300 µL of GCF-simulating medium) was inoculated, and they were incubated for 24 h at 37 °C under anaerobic conditions. The discs with the newly formed biofilm were then washed with PBS, a suspension of *P. gingivalis* and *F. nucleatum* (1:1 ratio in a total volume of 300 µL of GCF-simulating medium) was inoculated, and they were incubated for 14 days at 37 °C under anaerobic conditions, with replacement of the GCF medium every 48 h.

### 2.4. Confocal Laser Scanning Microscopy (CLSM)

A confocal microscope (Zeiss Confocal LSM710, Carl Zeiss Microscopy GmbH, Jena, Germany) was used to visualize the distribution of live and dead bacteria throughout the mature biofilm. A LIVE/DEAD viability kit (Life Technologies, Waltham, MA, USA) was used for sample staining. A total of 50 μL of the dye mixture (100 μL of SYTO 9 + 100 μL of propidium iodide + 800 μL of DDW) was added to each of the samples, which were then incubated in the dark at room temperature for 20 min. After incubation, the samples were rinsed with PBS, and 50 μL of mounting oil was added. Red fluorescence was measured under the microscope at 570 nm and green fluorescence at 520 nm, with objective lenses of ×2.5 and ×10. The stained biofilms were analyzed with ImageJ software (version 1.49g).

### 2.5. qPCR

DNA quantification of each bacterial species in the mature biofilm, grown for 14 days, was performed via quantitative polymerase chain reaction (qPCR), as described in detail by Periasamy and Kolenbrander (2009) and Kolenbrander et al. (2010) [10,28]. Biofilm quantification with qPCR was based on species-specific 16s rRNA gene primers (Hy Laboratories, Park TAMAR, Rehovot, Israel). The reaction mix contained a total volume of 20 μL, which consisted of SYBR green, forward and reverse primers, DEPC, and the examined sample. A Bio-Rad instrument (CFX96 Real-Time System, Bio-Rad, Hercules, CA, USA) was used for the qPCR reaction. The primers specific for *P. gingivalis* were F, TGGGTTTAAAGGGTGCGTAG, and R, CAATCGGAGTTCCTCGTGAT; the primers specific for *A. naeslundii* were F, GGACGGGTGAGTAATGCTTG, and R, CCCTTACCCCACCAACTACC; the primers specific for *S. sanguinis* were F, CGACGATACATAGCCGACCTGAG, and R, TCCATTGCCGAAGATTCCCTACTG; the primers specific for *F. nucleatum* were F, CTTAGGAATGAGACAGAGATG, and R, TGATGGTAACATACGAAAGG. The annealing temperature was 57 °C.

### 2.6. Cell Culture

The osteoblast-like cell line Saos-2 (kindly provided by the Periodontology Department, Hadassah Medical Center, Jerusalem, Israel) and the macrophage-like RAW 264.7—a murine leukemic monocyte cell line—were grown in Dulbecco’s modified Eagle’s medium (DMEM, Sigma-Aldrich, St Louis, MO, USA) supplemented with 15% fetal calf serum, 1% glutamine (2 mmol/L), and 1% penicillin/streptomycin (25 mg/mL), in 96-well culture plates. The osteoblast-like cells were harvested with trypsin–ethylenediaminetetraacetic acid (EDTA), while the macrophage-like cells were harvested by scraping, then seeded in the culture wells containing the discs at a density of 75,000 cells/200 mL/well, and cultured (5% CO_2_ and 37.5 °C). After 48 h, the cells were harvested for XTT analysis and quantified with a cell proliferation colorimetric assay (Biological Industries, Beit HaEmek, Israel). The ALP activity of the osteoblast-like cells was determined with a commercial kit (Sigma-Aldrich, St. Louis, MO, USA), and the activity of macrophage-like cells was assessed by the level of TNF-α cytokine in an enzyme-linked immunosorbent assay (ELISA) kit (MaxiSorp; Nunc, Naperville, IL, USA), according to the manufacturer’s instructions.

### 2.7. Statistical Analysis

All of the experiments were repeated at least three times and performed in triplicate. The results are expressed as the mean + SD of at least three independent experiments. The differences in mean values were analyzed using the *t*-test and ANOVA. A *p*-value of < 0.05 was considered statistically significant.

## 3. Results

**Figure 1:**
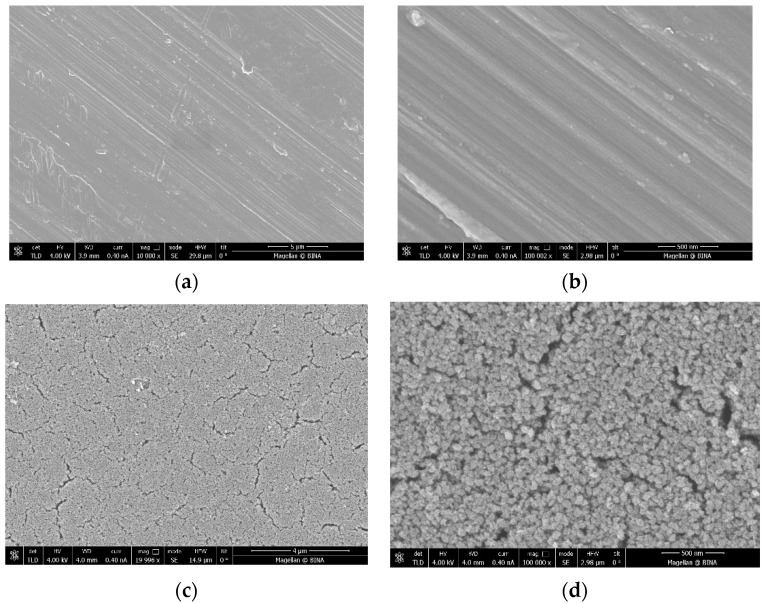
Images taken by high-resolution scanning microscopy (HRSEM) of (**a**,**b**) a disc before the coating process under low (10 K) and high (100 K) magnification, respectively, and (**c**,**d**) a coated disc under low (20 K) and high (100 K) magnification, respectively.

The ICP data indicate that the coated amount of Zn–CuO on the disc surface is ~0.8 wt% when the molar ratio of Cu:Zn is 8:1. These results are similar to those previously reported by Malka et al. (2013) [23]. The morphology of the coating was studied by high-resolution scanning microscopy (HRSEM), and is depicted in Figure 1. Low-magnification images of uncoated (Figure 1a) and coated discs (Figure 1c) clearly show the changes in the surface morphology. The coated disk is homogeneously coated with particles. Figure 1b,d show higher magnification of uncoated and coated disks, respectively. Figure 1d depicts the morphology of the particles, which have a spherical shape.

**Figure 2:**
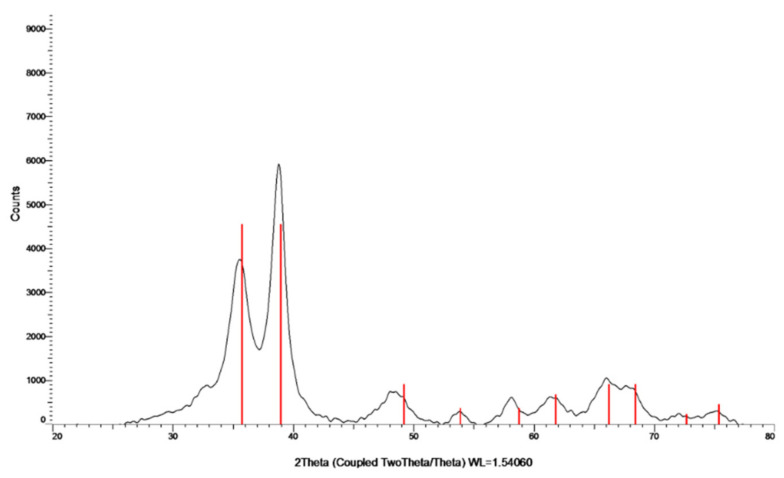
XRD pattern of Zn–CuO NPs; the red lines belong to the monoclinic CuO phase.

The crystalline nature of the formed particles was studied by XRD (Figure 2). The analysis was carried out on the collected and washed powder. The XRD pattern indicates the formation of a crystalline phase. The peaks at 2θ = 32.47, 35.49, 38.68, 48.65, 58.25, and 61.45 are slightly shifted in comparison to the (110), (−111), (111), (−202), (202), and (−113) reflection lines of monoclinic CuO particles, respectively, which also indicates the oxidation state of Cu as +2. The shift in the diffraction lines is assigned to the doping of Zn ions into the CuO lattice. With respect to the mechanism of Zn doping, for more details please see the work of Malka et al. (2013) [23].

**Figure 3:**
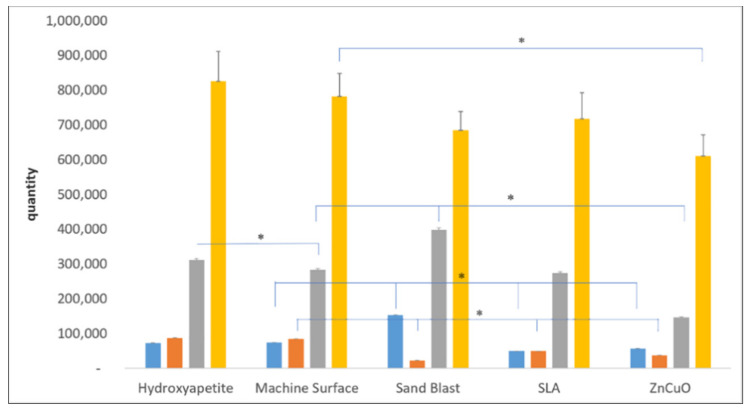
qPCR quantification of bacteria in multispecies biofilms grown on machined, sandblasted, SLA, and ZnCuO-coated titanium surfaces and hydroxyapatite discs after 14 days. Number of each bacterial species: *P. gingivalis* (blue columns), *F. nucleatum* (orange columns), *S. sanguis* (gray columns), and *A. naeslundii* (yellow columns), expressed as the mean + SD (*n* = 9; asterisk indicates *p* < 0.05).

Quantification of each bacterial strain in the four-species biofilm grown on the various disc surfaces after 14 days of incubation showed relatively small numbers of *P. gingivalis* and *F. nucleatum* on all of the disc surfaces (Figure 3). The ZnCuO-coated surfaces demonstrated a significant reduction in all of the species in the biofilm compared with the control machined and hydroxyapatite surfaces. The numbers of *F. nucleatum* on the sandblasted, SLA, and ZnCuO-coated surfaces were 0.26-, 0.58-, 0.44-fold lower than that on the control machined surface, respectively. The numbers of *P. gingivalis* and *S. sanguis* on the sandblasted surfaces were 2.08-fold and 1.45-fold higher than that on the machined control surface, respectively, whereas the number of *P. gingivalis* on the SLA surface was 0.32-fold lower than that on the control.

**Figure 4:**
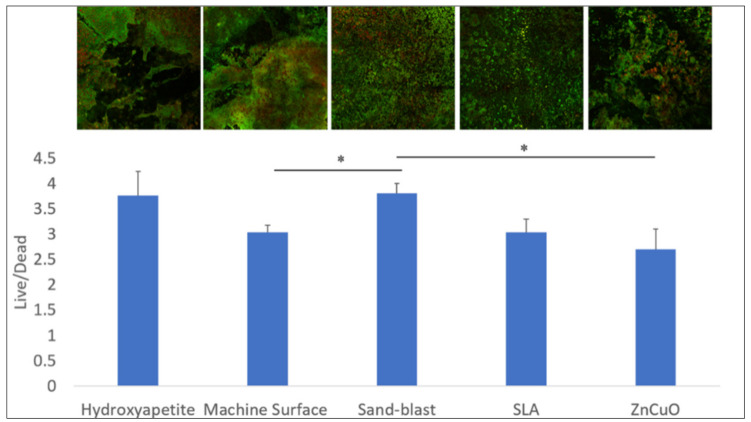
Confocal microscopy analysis of the live/dead assay in the biofilms grown on machined, sandblasted, SLA, and ZnCuO-coated titanium surfaces and hydroxyapatite discs after 14 days. Images showing staining of live bacteria in green and dead bacteria in red; bars showing the ratio of live to dead bacteria, expressed as the mean + SD (*n* = 4; asterisk indicates *p* < 0.05).

The bacterial viability in the multispecies biofilms grown on the various disc surfaces after 14 days of incubation is shown in Figure 4. The sandblasted surface showed a higher live/dead bacteria ratio compared with that of the machined and the ZnCuO-coated surfaces.

**Figure 5:**
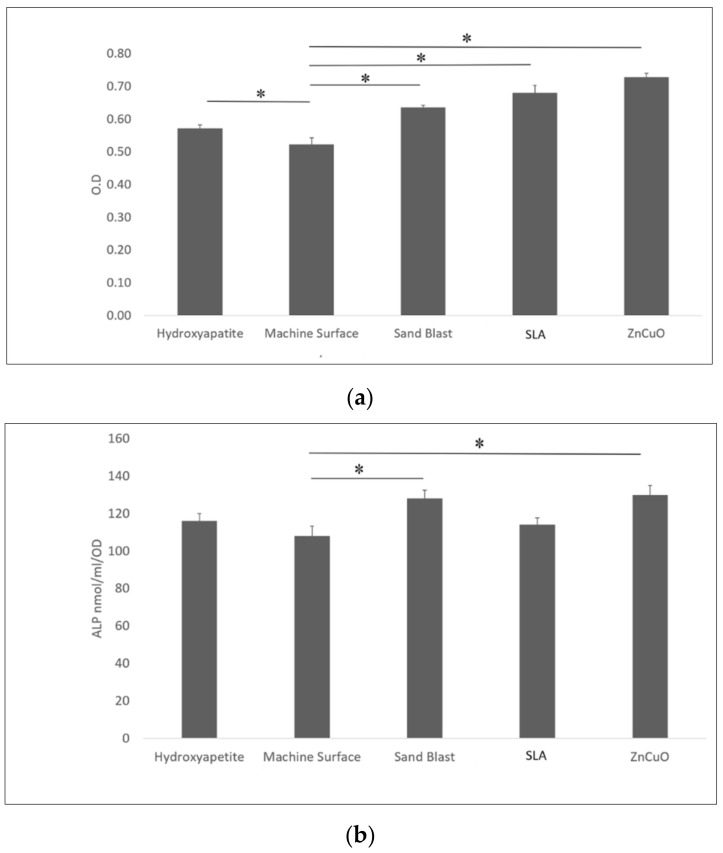
Osteoblast-like cells seeded in culture wells on machined, sandblasted, SLA, and ZnCuO-coated titanium surfaces and hydroxyapatite discs, after 48 h incubation: (**a**) cell proliferation (XTT assay); (**b**) alkaline phosphatase (ALP) activity, expressed as the mean + SD (*n* = 9; asterisk indicates *p* < 0.05).

Osteoblast-like cell proliferation (XTT assay) on hydroxyapatite, sandblasted, SLA, and ZnCuO-coated surfaces was 1.1-, 1.23-, 1.31-, 1.4-fold higher than that on the machined control surface, respectively (Figure 5a). ALP activity was significantly higher in the cells cultured on the ZnCuO-coated and sandblasted surfaces than on the machined surface (1.2- and 1.19-fold, respectively) (Figure 5b).

**Figure 6:**
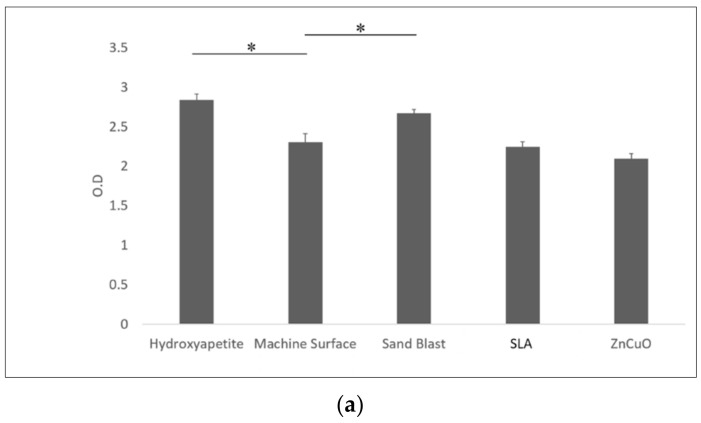

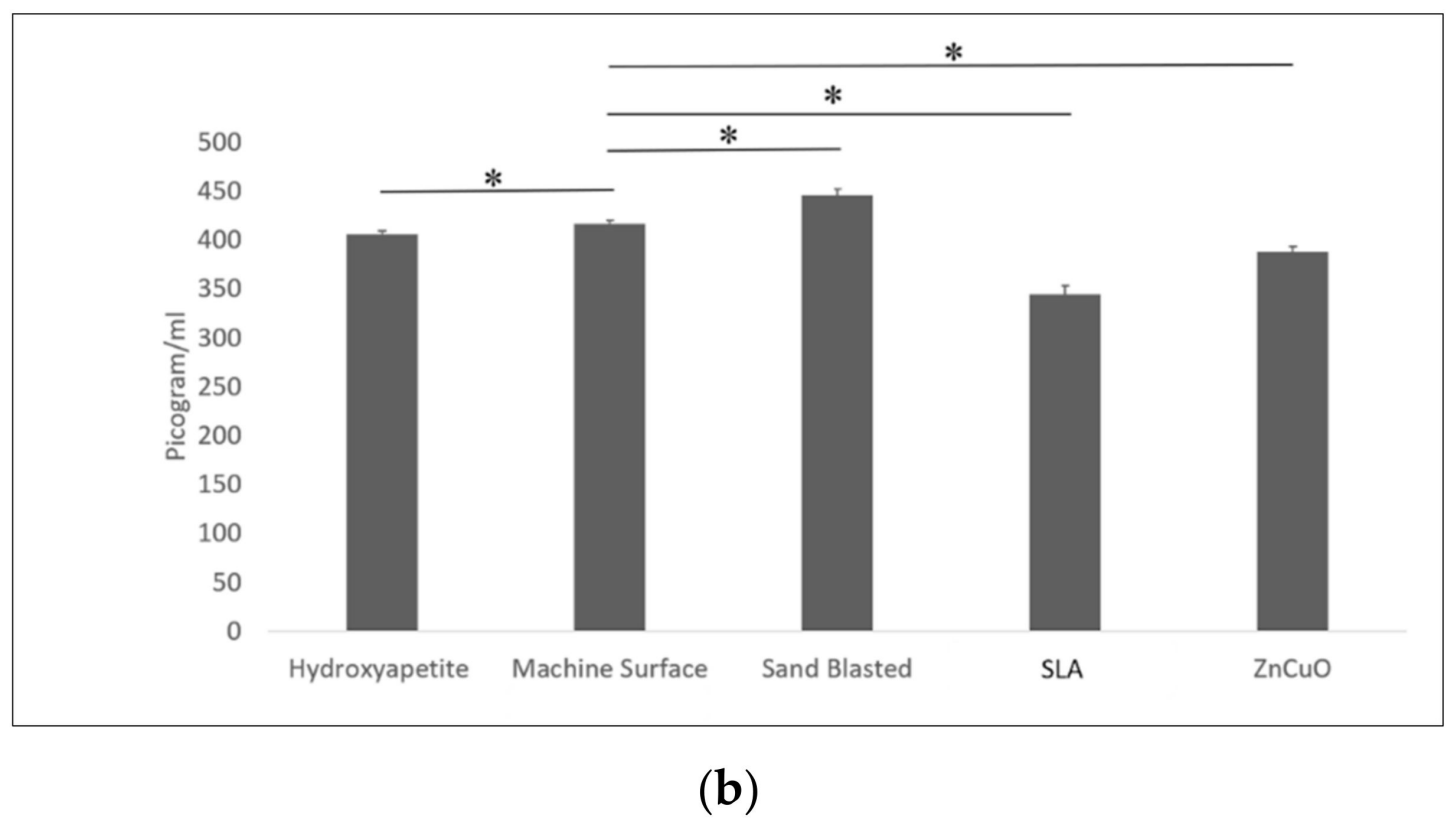
Macrophage-like cells seeded in culture wells on machined, sandblasted, SLA, and ZnCuO-coated titanium surfaces and hydroxyapatite discs, after 48 h incubation: (**a**) cell proliferation (XTT assay); (**b**) level of TNF-α cytokine produced by the cells, expressed as the mean + SD (*n* = 9; asterisk indicates *p* < 0.05).

Macrophage-like cell proliferation (XTT assay) was significantly higher on the sandblasted and hydroxyapatite surfaces than on the machined surface (1.16- and 1.23-fold, respectively) (Figure 6a). TNF-α cytokine production was significantly higher in cells cultured on the sandblasted surface compared with the hydroxyapatite, machined, SLA, and ZnCuO-coated surfaces (1.1-, 1.07-, 1.3-, and 1.15-fold, respectively) (Figure 6b).

## 4. Discussion

The findings of the present study show that ZnCuO-coated titanium surface discs may reduce biofilm accumulation, enhance osteoblast activity, and modulate the immune reaction. Our aim was to evaluate the potential of a novel ZnCuO nanoparticle coating of a titanium surface suggested for dental implants. Therefore, we designed a relatively simple and, thus, repeatable and controlled model composed of an in vitro four-species biofilm, early and later colonizers, and two representative host cells to closely simulate the multiple peri-implant conditions in the oral cavity.

Dental implant osseointegration is a complex phenomenon dependent on several factors and characterized by a long sequence of events. Although it is well known that micro-rough or nano-rough implant surfaces are beneficial for accelerated osseointegration, their long-term success is debatable, as there is a higher risk of peri-implantitis due to a higher propensity for plaque accumulation [29]. Our findings indicate that ZnCuO nanoparticle coatings of titanium implants have an inhibitory effect on the accumulation of all of the bacterial strains in the biofilms tested compared with that of the control machined surface, although the live/dead bacterial ratio was similar to that of the control. On the other hand, the sandblasted surface appeared to harbor relatively more live bacteria in the biofilm compared with the less rough surfaces (the machined and ZnCuO-coated surfaces). This is consistent with previous studies indicating that implant surface roughness is the main feature favoring biofilm development [6,30]. Moreover, rough surfaces are difficult to clean, resulting in rapid regrowth of the biofilm by multiplication of the remaining species, rather than by recolonization [31]. However, clinical observations showed no significant differences in plaque and bleeding indices related to implant surface topography [32], and no evidence for correlation between rough surface implants and the incidence of peri-implantitis [33].

The bacterial composition of our biofilm model grown for 14 days on different surfaces showed relatively low amounts of the periopathogens *F. nucleatum* and *P. gingivalis*, the dominant bacteria inhabiting the biofilm being *S. sanguis* and *A. naeslundii*. Although the biofilm grown on the ZnCuO-coated surface showed a decrease in all bacterial species, the sandblasted surface displayed an increased number of *P. gingivalis* and *S. sanguis* and a decrease in *F. nucleatum* in the biofilm compared with that of the machined surface.

The ZnCuO-coated surface exhibited a non-specific anti-biofilm effect, which was similar to the results obtained by other studies that used different coating strategies [14,19]. The anti-biofilm effect could be explained by the mechanism involved in intracellular ROS generation induced by the ZnCuO nanoparticles, which tended to adhere to bacterial cell surfaces and disrupt cell membrane integrity [24,25]. Electrochemical MAO treatment of Ti resulted in the formation of Ag-, Cu-, and Zn-incorporated oxide layers that showed both antibacterial properties against various bacteria and cytotoxicity in a dose-dependent manner [21]. The bactericidal effect could be attributed to a trap-killing system of the titanium dioxide layer on the surface [21]. However, suitable concentrations of elements may lead to a dual-functionalization of the Ti surface. This emphasizes the limitation of the possible modifications in the coating’s ability to reduce the biofilm accumulation while enhancing osseointegration.

Our findings showed that the osteoblast-like cells cultured on the ZnCuO-coated surfaces as well as on the other modified titanium surfaces tended to exhibit greater proliferation and higher ALP activity when on ZnCuO-coated and sandblasted surfaces, compared with the machined surface. Indeed, previous studies reported that the ALP activity of osteoblast-like cells was higher on rough surfaces [34,35]. Although the ZnCuO-coated surface was less rough than the sandblasted surface, our findings indicate that osteoblast activity is increased not only by surface topography, but also by biochemical properties, which may influence osteoblast proliferation, differentiation, matrix synthesis, and growth factor production [36,37]. Various studies have shown that Zn-based biomaterials are promising in bone regeneration [38,39]. Indeed, Zn facilitated osteoblast bone regeneration and inhibited osteoclastic bone resorption [38], and also stimulated cell proliferation and differentiation, as well as protein synthesis, in osteoblastic cells [39]. Our results are in agreement with these studies.

A similar proliferative effect of the sandblasted rough surface was observed on the macrophage-like cells, consistent with previous studies showing that these cells preferentially grow well on rough surfaces [40,41]. Macrophages play an important role in directing the early events in tissue healing following implant placement. Their appearance prior to bone formation indicates that macrophage cytokine production might contribute to the process of subsequent bone formation [42]. TNF-α is known to play an important role in bone formation and repair, and also negatively regulates the production of bone matrix proteins and alkaline phosphatase, along with osteoblast differentiation [43,44]. In the present study, the sandblasted rough surface showed an increased level of TNF-α—i.e., a higher activity of macrophage-like cells—and lower activity in the SLA and ZnCuO-coated surfaces compared with the control machined surface. Our observations are consistent with other reports demonstrating the ability of titanium surface topography to activate macrophage cytokine expression [45]. On the other hand, the bioactive properties of ZnCuO nanoparticles may also affect the macrophage-like cells, rather than the surface topography. The toxicity of ZnO nanoparticles to immune cells involved in ROS generation [46,47] suggests that ZnO nanoparticles may function as immunotoxicants [48].

## 5. Conclusions

In conclusion, it appears that the new ZnCuO nanoparticle coating of the titanium surface suggested for dental implants has anti-biofilm characteristics, with the desired effects on host cell activity and proliferation. The ZnCuO nanoparticle coating of titanium appears to be a promising surface modification for dental implant applications in the complex battle against peri-implantitis. Nevertheless, the release of metal ions—especially Cu ions—might cause long-term cytotoxicity or side effects [18]. Thus, further studies are needed to investigate the coating’s stability when in contact with bodily fluids, and to evaluate its safety under the conditions of the oral cavity [17]. In addition, it seems to be interesting to examine the feasibility of this novel sonochemical strategy for nanocoating of dental implant materials aside from titanium—such as zirconia and polyether ether ketone (PEEK) [14]—as well as for other dental applications to improve oral health.

## Data Availability

Not applicable.
